# Evaluation of In Vivo Antidiarrheal Activities of 80% Methanol Extract and Solvent Fractions of Peels of *Colocasia esculenta* (Araceae)

**DOI:** 10.1155/2024/2728282

**Published:** 2024-07-12

**Authors:** Temesgen Obsa Terfa, Fekadu Abera Kebede, Monas Kitessa Beyene, Tilahun Tesfaye Abebe, Abebe Basazn Mekuria, Jibril Seid Yesuf

**Affiliations:** ^1^ Department of Medicine College of Medicine and Health Sciences Jigjiga University, P. O. Box 1020, Jigjiga, Ethiopia; ^2^ Department of Nursing College of Health Sciences Oda Bultum University, Chiro, Ethiopia; ^3^ School of Pharmacy College of Health and Medical Sciences Haramaya University, P.O. Box 138, Dire Dawa, Ethiopia; ^4^ Department of Pharmacy College of Medicine and Health Sciences Ambo University, P.O. Box 251, Ambo, Ethiopia; ^5^ Department of Pharmacology School of Pharmacy College of Medicine and Health Sciences University of Gondar, Gondar, Ethiopia

## Abstract

**Background:**

Diarrhea is the common gastrointestinal disorder accounting for 2.5 billion episodes and 1.5 million deaths annually. Limitations and inaccessibility of currently available medications are the main problem associated with treatment of diarrhea. Hence, medicinal plants are usually preferred to manage diarrhea because they may contain constituents with high activity and fewer side effects. Even though the dose, safety, and efficacy of *Colocasia esculenta* (L.) Schott are not substantiated scientifically, several societies use it for the treatment of diarrhea.

**Objective:**

This study was targeted at exploring the in vivo antidiarrheal activities of 80% methanol extract and solvent fractions of peels of *Colocasia esculenta* (L.) Schott in Swiss albino mice.

**Methods:**

The plant was collected and extracted with 80% methanol, followed by fractionation with distilled water, chloroform, and diethyl ether. Castor oil-induced diarrhea, enteropooling, and motility tests were used to evaluate antidiarrheal activity. The test groups received graded doses of 100 mg/kg, 200 mg/kg, and 400 mg/kg. Negative controls received 10 ml/kg of 2% Tween 80 while positive controls received loperamide (3 mg/kg) or atropine (5 mg/kg).

**Results:**

The crude and solvent fractions of the plant extract have induced significant effects in reduction of the number and weight of wet stools at all tested doses. However, delay in onset of diarrhea was observed only at 400 mg/kg (*P* < 0.001) for both crude extract and solvent fractions. In antienteropooling test, 80% methanol extract and solvent fractions have significantly reduced the weight and volume of intestinal contents, especially at 200 mg/kg and 400 mg/kg. Regarding the antimotility test, the crude extract reduced motility at all tested doses, whereas the solvent fractions reduced intestinal motility mainly at 400 mg/kg (*P* < 0.001).

**Conclusion:**

The study has revealed that the 80% methanol extract and solvent fractions of the plant possess antidiarrheal activities supporting the traditional antidiarrheal claims of the plant.

## 1. Introduction

Diarrhea is a gastrointestinal disorder characterized by fecal urgency and incontinence. It causes an increase in the frequency, weight, and/or fluid content of the stool [1, 2]. Diarrhea can be caused by a variety of substances, including medications, poisons, infections, gastrointestinal illnesses, poorly absorbable materials, and issues with inflammation and dysmotility in the GI tract [3]. It is estimated to be responsible for 3.6% of the world's illness burden and almost 8% of all pediatric deaths. Diarrhea in children under five causes around 2.5 billion episodes and 1.5 million fatalities worldwide each year. Diarrhea is more common in underdeveloped nations and accounts for over 21 percent of mortality in children under five [4]. Wash and immunizations are the two main ways to avoid diarrhea [5, 6]. Reversing or preventing dehydration and lowering the morbidity and mortality linked to it are the main objectives of treating diarrhea [7]. Supportive therapy, antidiarrheal therapy, and empirical antibiotic therapy are the three primary methods used to treat diarrhea. The management of diarrhea can benefit greatly from currently prescribed medications, but they are also associated with a number of negative side effects, including bronchospasm, vomiting, intestinal blockage, constipation, dependency, drug resistance, and superinfections [8–10]. Despite advancements in modern medicine, many individuals, particularly in developing nations, depend on medicinal plants for healthcare, including diarrhea treatment. Traditional healers rely on ancestral knowledge, lacking precise information on dosage, safety, and chemical composition. It is crucial to scientifically validate the efficacy, safety, and usefulness of active constituents in medicinal plants to ensure their reliable integration into medical practices [11].

Traditional healers have long relied on a variety of medicinal plants with potential antidiarrheal properties, although the scientific evaluation of their effectiveness remains limited [12]. In the context of Ethiopian traditional medicine, numerous traditional remedies have been recorded, including the use of *Colocasia esculenta* (L.) Schott, commonly known as “Godere,” “Yitri,” or “Wuhayinkash.” Dating back to ancient times, this herb has been known for its healing properties and has been employed to treat various ailments, such as asthma, arthritis, diarrhea, internal hemorrhage, neurological disorders, skin disorders, body ache, cancer, diabetes, and baldness [13, 14]. However, further scientific research is needed to fully understand and validate the therapeutic potential of this plant in treating diarrhea and other diseases. Therefore, this study aimed to investigate antidiarrheal activity of 80% methanol extract and solvent fractions of peels of *Colocasia esculenta* (L.) Schott in Swiss albino mice. The study's findings offer a valuable foundation for the development of innovative antidiarrheal drugs, aiming to overcome limitations associated with existing medications. Furthermore, the scientific exploration of this plant's antidiarrheal properties not only validates and safeguards traditional wisdom but also fosters cultural diversity and preserves indigenous medicinal practices for the benefit of future generations. These findings serve as a guiding compass for future researchers, students, healthcare providers, and traditional medicine practitioners, facilitating their endeavors in advancing knowledge and improving patient care.

## 2. Materials and Methods

### 2.1. Plant Material

In March 2021, the peels of *Colocasia esculenta* were gathered from Deko Kebele, Wonago Woreda, located in the Southern Nation, Nationalities, and Peoples Region of South Ethiopia. The plant's authenticity was confirmed by a Botanist, Mr. Abiyu Eniyew Molla, affiliated with the Department of Biology at the College of Natural and Computational Sciences, University of Gondar. A voucher specimen with the reference number 001/TOT/2021 was preserved for future reference purposes.

### 2.2. Experimental Animals

The study adhered to established protocols for the care and use of laboratory animals [15]. A total of 293 healthy adult Swiss albino mice, both male and female, aged 8–12 weeks and weighing between 20 and 30 g, were used throughout the entire duration of the experiment. These mice were procured from the animal unit of the Department of Pharmacology at the University of Gondar.

The animals were housed in plastic cages, accommodating up to six mice per cage, with wood chip bedding changed every three days. They were provided with adequate food and water, and the lighting conditions followed a natural cycle of 12 hours on and 12 hours off. This was done to mimic the natural light-dark cycle experienced by the animals in their natural habitat, with light during the day and darkness during the night. This approach may help to regulate their circadian rhythms and ensure a more conducive environment for their well-being and normal physiological functioning. Prior to commencing the experiment, the mice were acclimatized to the laboratory environment for a week [16].

### 2.3. Extraction Process

The fresh peels of *Colocasia esculenta* (L.) Schott were carefully washed and cut into small pieces. To protect plant's constituents from sunlight-induced damage, the smaller pieces were dried under shade. Once dried, the plants were coarsely powdered using a mortar and pestle. Approximately 1700 grams of the plant powder was utilized for extraction. The powder was then subjected to cold maceration in an Erlenmeyer flask, mixed with 80% methanol in a 1 : 5 (w/v) ratio, and left to soak at room temperature for 72 hours with periodic shaking [17]. Following the initial extraction, the residue was filtered through muslin cloth and Whatman No. 1 filter paper to separate the liquid from the solid material. To maximize the yield, the remaining solid material underwent two additional extraction cycles using fresh solvent. The resulting fluid extracts were combined, filtered, and subjected to evaporation at 40°C using a rotary evaporator. The concentrated extract was then transferred to a hot air oven to remove any remaining liquid content. Subsequently, the extract underwent a deep-freezing process for 24 hours and was subsequently dried into powder form using a vacuum freeze dryer. Finally, the powder was stored in a refrigerator at 4°C until it was ready for use [18]. The percentage yield of the extract was found to be 8.23% (w/w).

### 2.4. Fractionation Process

After confirming the safety and dose-dependent antidiarrheal activity of the crude extract, further fractionation was conducted. A total of 120 g of the crude extract was subjected to successive fractionation using diethyl ether, chloroform, and distilled water. To begin, the crude extract was suspended in 600 mL of distilled water at a 1 : 5 ratio. The suspension was then placed in a separatory funnel, and an equal volume of diethyl ether (600 mL) was added. The mixture was thoroughly mixed and allowed to settle until distinct layers formed, facilitating the extraction of diethyl ether-soluble constituents. The diethyl ether fraction (upper layer) was collected in a beaker, and this process was repeated two more times.

Following the diethyl ether fraction collection, an equal volume (600 mL) of chloroform was introduced to the remaining aqueous residue and thoroughly mixed. Once a clear layer formed, the chloroform layer at the bottom was carefully collected in a beaker. This process was repeated twice more to ensure maximum extraction. Both the diethyl ether and chloroform fractions were subjected to evaporation using a rotary evaporator, and the resulting fractions were stored in a refrigerator at 4°C. The remaining aqueous residue was also collected and evaporated at 40°C using a rotary evaporator. The resulting residue was then placed in a deep freezer, followed by drying in a lyophilizer to obtain the aqueous fraction. After complete drying, the fraction was stored in an airtight container in the refrigerator until it was ready for use in the subsequent tests [17].

### 2.5. Preliminary Phytochemical Screening Test

The 80% methanol extract and the various solvent fractions underwent standard screening tests to determine the presence of specific phytoconstituents. These tests included the screening for flavonoids, tannins, anthraquinones/steroids, glycosides, phenols, terpenoids, alkaloids, and saponins. The purposes were to identify and evaluate the phytochemical composition of the extract and fractions [17].

### 2.6. Acute Oral Toxicity Test

To assess acute oral toxicity, a total of five healthy, nonpregnant female Swiss albino mice, aged 8–12 weeks, were employed due to their heightened sensitivity [19]. The mice were acclimatized to the laboratory environment for one week prior to the experiment. Following a fasting period of 4 hours, the first mouse was administered a dose of 2 g/kg of the 80% methanol extract, in accordance with Organization for Economic Co-operation and Development (OECD) 425 guidelines. The mouse was then closely monitored for any physical or behavioral changes within a 24-hour period, with particular attention during the initial 4 hours. As the first mouse displayed no signs of toxicity, the remaining four mice were subsequently treated with the same dose and observed daily for 14 days for any indications of toxicity, such as loss of appetite, hair erection, lacrimation, tremors, convulsions, salivation, diarrhea, mortality, and other adverse effects [20].

### 2.7. Animal Grouping and Dosing

For the experiment, a healthy adult Swiss albino mouse, both males and females, aged between 8 and 12 weeks and weighing 20−30 g, was utilized. The study involved three models and included the 80% methanol extract as well as each of the solvent fractions. The mice were randomly assigned to five groups, each consisting of six mice for each model. Group I served as the negative control and received 10 mL/kg of 2% Tween 80 in all models. Group II, the positive control, received loperamide at a dose of 3 mg/kg (10 mL/kg) for the castor oil-induced diarrhea and intestinal fluid accumulation test. Atropine sulfate at a dose of 5 mg/kg was administered intraperitoneally as the positive control for the GI motility test. Groups III, IV, and V were treated with doses of 100 mg/kg, 200 mg/kg, and 400 mg/kg of the *Colocasia esculenta* (L.) Schott peel extracts, respectively [21, 22].

### 2.8. Antidiarrheal Activity Determination

#### 2.8.1. Antidiarrheal Activity on Castor Oil-Induced Diarrhea

A total of thirty Swiss albino mice, regardless of their sex, were selected and weighed before being randomly assigned to five groups, with six mice in each group. The mice underwent an 18-hour fasting period but had access to water. The dosing and grouping were carried out as described earlier. One hour after administration of the appropriate extract, each mouse was orally administered 0.5 mL of castor oil using a gavage technique. The mice were then individually placed in metabolic plastic cages lined with nonwetting transparent paper, which was changed whenever the mice defecated. Over a four-hour observation period, various parameters such as the onset of diarrhea, frequency of wet feces, total number of defecations, weight of wet and dry stool, and consistency of feces were recorded and compared to the control group. The percentage inhibition of diarrhea and defecations was calculated for both the crude extracts and solvent fractions [23–25].

(1) $$\begin{array}{c} \%\;I\text{nhibition of diarrhea}=\frac{M\text{ean number of wet stools of }\left(\text{control group}\_\text{treated group}\right)}{M\text{ean number of wet stools of control group}}*100,\\ \%\;I\text{nhibition of defecation}=\frac{T\text{otal number of feces in the }\left(\text{negative control}\_\text{treated group}\right)}{T\text{otal number of feces in the negative control}}*100. \end{array}$$

#### 2.8.2. Castor Oil-Induced Enteropooling

A group of thirty mice was randomly divided into five groups, with six mice in each group. The mice underwent an 18-hour fasting period but had access to water. Following the dosing procedure mentioned under animal grouping and dosing section, after one hour, each mouse was administered 0.5 mL of castor oil. An hour later, the mice were euthanized by cervical dislocation. The abdominal cavity was opened, and the small bowel was carefully dissected, isolated, and ligated at both the pylorus and cecum ends. The weight of the dissected small bowel was measured, and its contents were milked into a measuring cylinder to determine the volume. The weight of the intestine after milking was measured, and the weight of the intestinal content was calculated by subtracting the weight of the intestine before and after milking. Finally, the percent inhibition of intestinal secretion, by both volume and weight, was calculated for both the crude extract and the solvent fractions [26–28].

(2) $$\begin{array}{c} \%\text{of inhibition by using MWIC}=\frac{\text{MWICC}-\text{MWICT}}{\text{MWICC}}*100, \end{array}$$

where MWIC is the mean weight of intestinal content, MWICC is the mean weight of intestinal content of control group, and MWICT is the mean weight of intestinal content of test group, and

(3) $$\begin{array}{c} \%\text{of inhibition by using MVIC}=\frac{\text{MVICC}-\text{MVICT}}{\text{MVICC}}*100, \end{array}$$

where MVIC is the mean volume of intestinal content, MVICC is the mean volume of intestinal content of control group, and MVICT is the mean volume of intestinal content of test group.

#### 2.8.3. Gastrointestinal Motility Test by Activated Charcoal Suspension

A total of thirty mice underwent an 18-hour fasting period, with access to water, and were divided into five groups of six mice each, as previously mentioned. Following the animal grouping and dosing procedure, one hour after treatment with the extract and/or controls, the mice were challenged with 0.5 mL of castor oil. After thirty minutes, the mice received 0.5 mL of a 10% charcoal suspension in 2% Tween 80. Thirty minutes after the administration of the charcoal suspension, the mice were sacrificed, and their abdomens were opened to remove the intestine from the pylorus to the cecum. The intestine was then placed on white paper, and the length of the small intestine and the distance traveled by the charcoal suspension from the pylorus to the cecum were measured using a ruler. The peristalsis index, representing the percentage of the overall length of the small intestine that the charcoal meal had traversed, was calculated. Finally, the percent inhibition of intestinal transit was determined by comparing the mean distance traveled by the charcoal meal with the controls [28–31].

(4) $$\begin{array}{c} \text{Peristalsis index}\left(\text{PI}\right)=\frac{\text{distance traveled by the charcoal suspension}}{\text{total length of small intestine}}*100,\\ \%\text{ of inhibition}=\frac{\text{PI of negative control}-\text{PI of drug or extract treated}}{\text{ PI of negative control}}*100. \end{array}$$

#### 2.8.4. In Vivo Antidiarrheal Index (ADI)

The antidiarrheal index (ADI) was determined through the combination of data obtained from the castor oil-induced diarrhea, enteropooling, and gastrointestinal (GI) motility tests. The ADI was calculated using a specific formula, taking into account the results from these tests [22].

(5) $$\begin{array}{c} \text{ADI invivo}=\sqrt[{3}]{\text{DDT}*\text{GMT}*\text{IFA}}, \end{array}$$

where DDT = the delay in defecation time or diarrheal onset (as % of control), GT = the GI motility by charcoal travel reduction (as % of control), and IFA = the reduction in the intestinal fluid accumulation (as % of control).

(6) $$\begin{array}{c} \text{DDT}=\frac{O\text{nset of diarrhea in }a\text{ minute of the }\left(\text{ test}-\text{negative control}\right)\text{ group}}{O\text{nset of diarrhea in }a\text{ minute of the negative control group }}*100,\\ \text{GMT}=\frac{D\text{istance traveled by the charcoal marker of the }\left(\text{ negative control}\_\text{test}\right)\text{ group}}{D\text{istance traveled by the charcoal marker in the negative control group}}*100,\\ \text{IFA}=\frac{M\text{ean weight of wet stools of }\left(\text{the negative control}-\text{treated}\right)\text{ group}}{M\text{ean weight of wet stools of negative control group}}*100. \end{array}$$

### 2.9. Ethical Clearance

Prior to commencing the experimental activities, the research proposal was submitted to and approved by the Department Graduate Committee of Pharmacology at the University of Gondar. Additionally, ethical clearance was obtained from the same department, with a reference number of Sop4/103/2013. The handling and care of the experimental animals adhered to both local and international guidelines regarding the use, care, and welfare of laboratory animals.

### 2.10. Statistical Analysis

The results were presented as the mean ± standard error of the mean (SEM) and were subjected to statistical analysis using SPSS version 26.0. Group comparisons were evaluated using one-way analysis of variance (ANOVA), followed by a post hoc Tukey test. Statistical significance was considered for probability values below 0.05. The processed data were summarized and presented in tabular form.

### 2.11. Data Quality Assurance

Data were compiled, cleared, coded, and checked for completeness and accuracy before entering into SPSS version 26.0. Mice were assigned to each group through random selection to reduce bias.

## 3. Results

### 3.1. The Percentage Yield

A total of 1700 g of plant powder was subjected to extraction, resulting in the production of 140 g of crude extract. The crude extract (120 g) was further fractionated using solvents of increasing polarity, namely, diethyl ether, chloroform, and distilled water. The percentage yield was then calculated for each fraction. The results indicated a percentage yield of 8.23% (140 g) for the crude extract, 46.7% (56 g) for the aqueous fraction, 26.67% (32 g) for the chloroform fraction, and 9.16% (11 g) for the diethyl ether fraction.

### 3.2. Preliminary Phytochemical Screening

Table 1 shows the outcomes of a preliminary phytochemical screening conducted on both the crude extract and solvent fractions of the plant. The screening revealed the potential presence of various bioactive compounds, including flavonoids, tannins, anthraquinones, steroids, glycosides, phenols, terpenoids, alkaloids, and saponins (Table 1).

### 3.3. Acute Oral Toxicity Test

Following the oral administration of a single dose of 2000 mg/kg of the hydromethanolic peel extract of *Colocasia esculenta* (L.) Schott, no deaths or observable signs of toxicity were reported within a 24-hour period. Additionally, throughout a 14-day observation period, no indications of toxicity such as loss of appetite, hair erection, lacrimation, tremors, convulsions, salivation, or diarrhea were observed. These findings suggest that the plant extract possesses a substantial safety margin and can be considered safe for use.

### 3.4. Effects of 80% Methanol Extract of the Peels of *Colocasia esculenta* (L.) Schott on Castor Oil-Induced Diarrheal Model

During a four-hour observation period following the administration of castor oil, the three doses (100, 200, and 400 mg/kg) demonstrated a noteworthy decrease in the number of wet stool (*P* < 0.001), total stool (*P* < 0.001), weight of wet stool (*P* < 0.001), and fluid content of stool (*P* < 0.001) compared to the negative control. However, it was observed that only the dose of 400 mg/kg (*P* < 0.01) significantly delayed the onset of diarrhea.

In contrast, the dose of 400 mg/kg exhibited a significant difference in the delay of diarrhea (*P* < 0.05), total number of wet stool (*P* < 0.05), and weight of wet stool (*P* < 0.05) when compared to the 100 mg/kg dose. The percent inhibition of diarrhea was found to be 44.03%, 60.58%, and 65.46% at the doses of 100, 200, and 400 mg/kg, respectively. In comparison, loperamide exhibited a percent inhibition of 78.23%. Similarly, the percent inhibition of defecation was 44.57%, 53.47%, and 63.69% at the doses of 100, 200, and 400 mg/kg, respectively, as presented in Table 2.

### 3.5. Effects of 80% Methanol Extract on Castor Oil-Induced Enteropooling

Upon dissecting and measuring the weight of small intestinal contents, all three doses (100 mg/kg, 200 mg/kg, and 400 mg/kg) demonstrated a significant decrease (*P* < 0.001) compared to the negative control. The percent inhibition of small intestinal fluid accumulation, assessed by weight, was 34.5%, 42.3%, and 60.4% at the doses of 100 mg/kg, 200 mg/kg, and 400 mg/kg, respectively. Loperamide exhibited a percent inhibition of 65.7% in the same regard.

In terms of the volume of small intestinal contents, all three doses showed a significant difference (*P* < 0.001) compared to the negative control. Additionally, the doses of 200 mg/kg and 400 mg/kg demonstrated a significant difference (*P* < 0.05) in comparison with the 100 mg/kg dose. The percent inhibition of small intestinal secretion, measured by volume, was 41%, 57%, and 66.4% at the doses of 100 mg/kg, 200 mg/kg, and 400 mg/kg, respectively, while the positive control exhibited a percent inhibition of 72.4%, as illustrated in Table 3.

### 3.6. Effects of 80% Methanol Extract on Castor Oil-Induced Gastrointestinal Motility

All doses of the crude extract from the plant exhibited a significant reduction (*P* < 0.001) in the peristalsis index compared to the negative controls. Furthermore, the dose of 400 mg/kg demonstrated a significant decrease (*P* < 0.001) in the peristalsis index when compared to the 100 mg/kg dose. The percent inhibition of intestinal transit of the charcoal meal through the gastrointestinal tract (GIT) was found to be 18.2%, 22.3%, and 38.9% at the doses of 100 mg/kg, 200 mg/kg, and 400 mg/kg, respectively. In comparison, the positive control, atropine (5 mg/kg), exhibited a percent inhibition of 64.4%, as depicted in Table 4.

### 3.7. Effects of Solvent Fractions of the Plant on Castor Oil-Induced Diarrheal Model

In the castor oil-induced diarrheal model, all three fractions significantly reduced the total number of wet stool and total stool at doses of 100 mg/kg (*P* < 0.001), 200 mg/kg (*P* < 0.001), and 400 mg/kg (*P* < 0.001) when compared to the negative control. Both the aqueous and chloroform fractions significantly reduced the weight of wet stool at 200 mg/kg (*P* < 0.001) and 400 mg/kg (*P* < 0.001), while the diethyl ether fraction only showed a reduction at 400 mg/kg (*P* < 0.001) compared to the negative control. All solvent fractions produced a significant delay in the onset of diarrhea and reduction in the fluid content of the stool, but only at a dose of 400 mg/kg compared to the negative control, with varying levels of significance. The percent inhibition of diarrhea for the aqueous fraction was 31.67%, 44.86%, and 55% at doses of 100 mg/kg, 200 mg/kg, and 400 mg/kg, respectively, while the standard drug loperamide exhibited a percent inhibition of 70.69% (Table 5).

The chloroform fraction did not exhibit significant activity at doses of 100 mg/kg and 200 mg/kg in terms of the delay in onset of diarrhea and fluid content of the stool compared to the negative control. However, it did show a significant difference in all tested parameters compared to the positive control. The percent inhibition of diarrhea by the chloroform fraction was 30.75%, 40.85%, and 57.73% at doses of 100 mg/kg, 200 mg/kg, and 400 mg/kg, respectively. Similarly, the diethyl ether fraction did not demonstrate significant activity at doses of 100 mg/kg and 200 mg/kg in relation to the weight of wet stool, fluid content of the stool, and delay in onset of diarrhea compared to the negative control. The percentage inhibition of diarrhea by the diethyl ether fraction was 27.5%, 37.23%, and 55.94% at doses of 100 mg/kg, 200 mg/kg, and 400 mg/kg, respectively (Table 5). When comparing the efficacy among the doses of the solvent fractions, the highest dose of 400 mg/kg exhibited significant activity in all tested parameters compared to doses of 100 mg/kg and 200 mg/kg.

### 3.8. Effects of Solvent Fractions on Castor Oil-Induced Enteropooling Model

**Figure 1:**
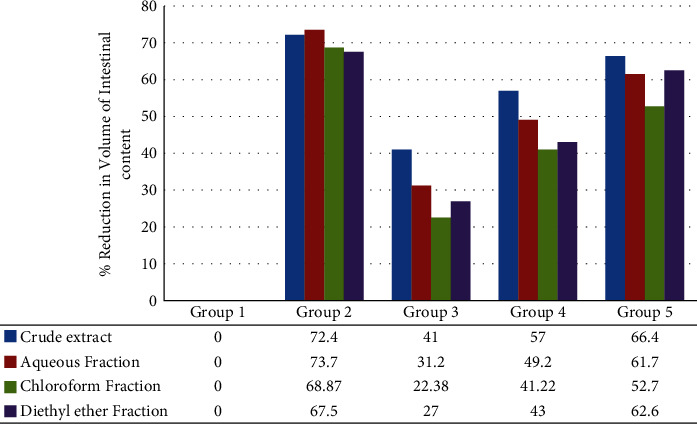
The effect of 80% methanol extract and solvent fractions of peels of *Colocasia esculenta* (L.) Schott on the percent inhibition in a volume of intestinal content. Group 1: negative control, group 2: positive control, group 3: 100 mg/kg, group 4: 200 mg/kg, and group 5: 400 mg/kg.

The aqueous fraction derived from the peels of *Colocasia esculenta* (L.) Schott exhibited significant reductions in both the mean weight and volume of intestinal contents at doses of 100 mg/kg (*P* < 0.05 for volume, *P* < 0.01 for weight), 200 mg/kg (*P* < 0.001), and 400 mg/kg (*P* < 0.001) compared to the negative control. The percentage inhibition of the weight of intestinal content was 29.68%, 45.36%, and 58.9% at doses of 100 mg/kg, 200 mg/kg, and 400 mg/kg, respectively. Similarly, the percentage inhibition by volume was 31.2%, 49.2%, and 61.7% at the corresponding doses. Moreover, the dose of 400 mg/kg demonstrated a significant difference compared to the 100 mg/kg dose of both the aqueous fraction and chloroform fraction in terms of the weight and volume of intestinal contents, with different levels of significance, as presented in Table 6 (Figure 1).

Similarly, the chloroform fraction also exhibited significant reductions in the weight and volume of intestinal contents at all doses: 100 mg/kg (*P* < 0.01 for weight), 200 mg/kg (*P* < 0.001 for weight, *P* < 0.01 for volume), and 400 mg/kg (*P* < 0.001) compared to the negative control. However, the 100 mg/kg dose did not show a significant effect on the volume of intestinal contents. The percent inhibition of weight and volume of intestinal contents was 22.2%, 39.6%, and 53.78% for weight, and 22.38%, 41.22%, and 52.7% for volume at doses of 100 mg/kg, 200 mg/kg, and 400 mg/kg, respectively (Figure 1).

Similarly, the diethyl ether fraction also significantly reduced the weight and volume of intestinal contents (*P* < 0.001) compared to the negative control. The percent inhibition of weight and volume of intestinal contents was 26.7%, 41.2%, and 60.2% for weight, and 27%, 43%, and 62.6% for volume at doses of 100 mg/kg, 200 mg/kg, and 400 mg/kg, respectively (Table 6) (Figure 1).

The highest dose of 400 mg/kg of all solvent fractions demonstrated significant effects on the weight and volume of intestinal contents compared to the 100 mg/kg dose, with different levels of significance. The percent inhibition by both weight and volume decreased from 400 mg/kg to 100 mg/kg in all solvent fractions (Figure 1).

### 3.9. Effects of Solvent Fractions on Castor Oil-Induced Gastrointestinal Motility

The aqueous fraction of the plant extract significantly inhibited intestinal transit of charcoal meal at a dose of 400 mg/kg (*P* < 0.001) compared to the negative control, while the doses of 100 mg/kg and 200 mg/kg did not show a significant effect on intestinal motility. All three doses of the aqueous fraction demonstrated a significant difference (*P* < 0.001) in intestinal motility compared to the standard drug, atropine sulfate. The percentage inhibition of intestinal propulsion was 9%, 16.8%, and 31.8% for doses of 100 mg/kg, 200 mg/kg, and 400 mg/kg, respectively, whereas atropine exhibited a 65% inhibition (Table 7).

Similarly, the chloroform fraction showed a significant effect on intestinal transit of charcoal suspension at doses of 200 mg/kg (*P* < 0.05) and 400 mg/kg (*P* < 0.001), except for the 100 mg/kg dose. The percent inhibition of gastrointestinal motility was 7.43%, 13.63%, and 33.2% for doses of 100 mg/kg, 200 mg/kg, and 400 mg/kg, respectively.

Likewise, the diethyl ether fraction significantly affected intestinal propulsion of charcoal meal at a dose of 400 mg/kg (*P* < 0.01) compared to the negative control. The percent inhibition of intestinal transit of charcoal meal was 6.8%, 12.6%, and 27% for doses of 100 mg/kg, 200 mg/kg, and 400 mg/kg, respectively.

### 3.10. In Vivo Antidiarrheal Index

**Figure 2:**
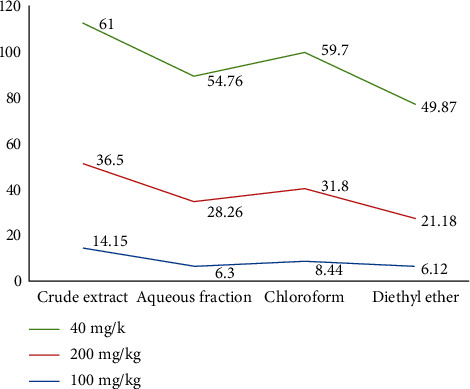
In vivo antidiarrheal index of crude extract and solvent fractions of peels of *Colocasia esculenta* (L) Schott.

The in vivo antidiarrheal indexes of both crude extract and solvent fractions of peels of *Colocasia esculenta* (L.) Schott have exhibited a variation among different doses of the plant extract as shown in Figure 2.

## 4. Discussion

The antidiarrheal properties of the peels of *Colocasia esculenta* (L.) Schott, a herbaceous plant traditionally used for managing diarrhea in various countries, were investigated in this study. Three different diarrhea models, including castor oil-induced diarrhea, enteropooling, and gastrointestinal motility tests, were conducted on Swiss albino mice to evaluate the claimed antidiarrheal activity.

In this study, the induction of diarrhea was achieved using castor oil, which contains ricinoleic acid as its active metabolite. Ricinoleic acid is known to stimulate the secretory process and increase intestinal motility by causing irritation and inflammation of the intestinal mucosal linings. This inflammation leads to the release of various inflammatory mediators, including prostaglandin (PGE-2), which can affect ion channels and disrupt normal physiological processes. Consequently, these inflammatory mediators, particularly PGE-2 and nitric oxide (NO), can inhibit glucose absorption, trigger inflammation of the intestinal mucosa, cause contraction of the intestinal smooth muscle, and disrupt ion channels, such as Na + -K + ATPase. These effects collectively contribute to an increase in intestinal secretion and motility [32, 33].

Previous studies [23, 27, 30] have indicated that various plant constituents such as saponins, flavonoids, alkaloids, terpenoids, phenols, anthraquinones, steroids, glycosides, and tannins are believed to be responsible for the antidiarrheal effects observed in medicinal plants. In the present study, phytochemical screening was conducted on the peels of *Colocasia esculenta* (L.) Schott, and it revealed the presence of these bioactive compounds in both the 80% methanol extract and solvent fractions. This finding supports the claim of the plant extract's antidiarrheal properties, although the specific constituents responsible for this effect have yet to be identified.

In the castor oil-induced diarrheal model, the frequency and weight of wet stool were given particular attention as they are commonly associated with diarrhea. The 80% methanol extract of plant's peels demonstrated significant activity at all tested doses in reducing the frequency and weight of wet stool. Notably, the dose of 400 mg/kg exhibited the highest inhibition of diarrheal stool (65.46%), indicating an increased effectiveness of the extract with higher doses.

The crude extract of the peels of *Colocasia esculenta* (L.) Schott demonstrated superior activity in inhibiting diarrheal stool compared to total defecation when compared to the negative control. Additionally, the crude extract was able to reduce the fluid content of the stool at all tested doses, indicating its ability to enhance colonic fluid absorption.

Regarding the delay of diarrhea onset, the dose of 400 mg/kg exhibited better activity compared to the standard drug loperamide. This may be attributed to the extract's essential absorptive properties and its ability to block chloride channels. However, the doses of 100 mg/kg and 200 mg/kg of the plant extract did not show a significant effect on delaying diarrhea. This could be due to the lower concentrations of phytochemicals present in these doses, which might not be sufficient to produce the desired therapeutic effect.

Overall, the crude extract of the peels of *Colocasia esculenta* (L.) Schott reduced all tested parameters at different doses, although the efficacy varied among the doses. The significant effects of the crude extract can be attributed to the presence of various phytoconstituents, particularly alkaloids, tannins, flavonoids, phenols, and terpenoids. These compounds inhibit prostaglandin (PG) secretion, fluid, and electrolyte secretion, while promoting absorption through different mechanisms. Furthermore, the plant's anti-inflammatory effects observed in in vivo studies could contribute to its enhanced efficacy [34].

Consistent with the previous findings, the solvent fractions of the plant exhibited a significant reduction in the total number of wet stool and the number of defecations at all tested doses. However, the solvent fractions only reduced the weight of wet stool at doses of 200 mg/kg and 400 mg/kg compared to the negative control. Regarding the onset of diarrhea and fluid content of the stool, all fractions demonstrated a significant reduction only at the dose of 400 mg/kg, indicating a dose-dependent effect of these parameters.

Furthermore, in the castor oil-induced diarrheal model, the 80% methanol extract was found to be more effective than the solvent fractions in terms of all tested parameters. This difference in effectiveness could be attributed to variations in the availability and concentrations of phytoconstituents among the different fractions. Among the solvent fractions, the aqueous fraction exhibited the highest effectiveness in inhibiting diarrhea, while the diethyl ether fraction was the least effective. However, the aqueous fraction showed the highest effectiveness in inhibiting defecations, and the chloroform fraction was the most effective in delaying the onset of diarrhea.

The less effective performance of the aqueous fraction in delaying diarrhea could be due to the absence of flavonoids, which play a crucial role in inhibiting prostaglandin (PG) release and gastrointestinal motility. Additionally, the available constituents in the aqueous fraction may not reach therapeutic concentrations, thereby limiting their intended effects. Conversely, the high effectiveness of the chloroform fraction in delaying diarrhea could be attributed to the presence of constituents such as alkaloids, flavonoids, and phenols, which are known to exhibit the required activity. Phenols can reduce intestinal secretion and enhance absorption through their antioxidant, anti-inflammatory, and antibacterial properties. Tannins regulate cystic fibrosis transmembrane conductance regulator (CFTR) and calcium-activated chloride channel (CaCC), thereby decreasing chloride secretions. Flavonoids have the potential to inhibit cyclooxygenase-1 (COX-1), cyclooxygenase-2 (COX-2), and lipoxygenase (LOX), thus reducing prostaglandin synthesis. They can also reduce intestinal motility by activating *α*2-adrenergic receptors, leading to sympathetic nerve activation and subsequent reduction in intestinal contractions. All of these factors contribute to the delay in the onset of diarrhea, decrease in fluid content of the stool, and reduce the frequency and weight of stool [35–38].

In the castor oil-induced enteropooling model, the crude extract at all tested doses exhibited significant reductions in the mean weight and volume of intestinal contents compared to the negative control. Notably, the dose of 400 mg/kg demonstrated the highest percentage of inhibition (60.4% for weight and 66.4% for volume) among all the doses tested. This suggests a clear dose-response relationship for the plant extract in this context.

Regarding the antienteropooling effects of the solvent fractions, all fractions demonstrated significant reductions in both the average weight and volume of intestinal contents, although there were variations among the different doses. However, the chloroform fraction exhibited the least activity in reducing intestinal fluid accumulation compared to the other fractions. This could be attributed to the absence of metabolites such as saponins, tannins, glycosides, and steroids, which are known to play a crucial role in antisecretory activities.

The crude extract of the plant showed significant antienteropooling effects at all tested doses, with the highest inhibition observed at the dose of 400 mg/kg. Similarly, the solvent fractions demonstrated reductions in intestinal fluid accumulation, although the chloroform fraction was comparatively less effective, possibly due to the absence of certain metabolites [35, 39]. The presence of tannins and steroids in the diethyl ether fraction could contribute to its higher effectiveness compared to the chloroform fraction. Steroids are known to induce antienteropooling effects through their anti-inflammatory properties, inhibiting the release of prostaglandin E-2 (PGE-2) and prostacyclin (PGI-2) from macrophages. On the other hand, tannins can inhibit secretion and reduce fluid accumulation by modulating the activity of cystic fibrosis transmembrane conductance regulator (CFTR) and calcium-activated chloride channel (CaCC) [40]. Other phytoconstituents present in the plant extract, such as alkaloids, glycosides, and anthraquinones, may also contribute to the antisecretory effects observed. These compounds have been reported to possess antibacterial, antifungal, anti-inflammatory, and antioxidant activities, which can collectively contribute to the inhibition of fluid secretion and accumulation in the intestines [38, 41].

The crude extract of *Colocasia esculenta* (L.) Schott, which contains a combination of phytochemicals, demonstrated superior efficacy compared to the solvent fractions in reducing intestinal fluid accumulation and motility. The aqueous fraction exhibited significant antisecretory effects, while the chloroform fraction showed the least effect in both parameters. The crude extract displayed a clear dose-response relationship, with higher doses being more effective in inducing antimotility effects, although the percentage inhibition of intestinal propulsion was lower compared to the standard drug atropine sulfate.

Among the solvent fractions, the chloroform fraction exhibited the highest activity in reducing intestinal motility. This may be attributed to the presence of intermediate polarity phytochemicals such as flavonoids, alkaloids, and phenols, which are known to impact peristalsis. However, it is important to note that the overall activity of the solvent fractions in inhibiting intestinal motility was lower than the standard drug. The presence and concentration of bioactive compounds, including tannins, terpenoids, flavonoids, phenols, and alkaloids, likely contribute to the regulation of peristalsis, with tannins reducing calcium influx and flavonoids activating *α*2-adrenergic receptors, resulting in decreased motility and prolonged transit time [35, 38]. In previous studies, it has been indicated that the presence of flavonoids, alkaloids, and phenols may contribute to antidiarrheal effects. These effects are believed to occur through various mechanisms, including the inhibition of arachidonic acid metabolism, promotion of fluid and electrolyte absorption, inhibition of secretion, and reduction of intestinal motility [42].

Hence, those abovementioned notions suggested another possible antidiarrheal mechanism of the plant extract that could be inhibition of intestinal motility via blockage of Ca^2+^ channel, inhibition of PG and NO release, hampering of Ach and 5-HT release, and/or preventing inflammation of intestinal mucosa [43]. Hence, a decrease in intestinal motility allows more time for absorption of intestinal contents.

The assessment of antidiarrheal activity in medicinal plants often relies on in vivo antidiarrheal indices (ADI), which consider multiple parameters, such as the onset of diarrhea, mean weight of wet stools, and distance traveled by charcoal suspension [30]. In this study, the crude extracts and diethyl ether fraction displayed the highest and lowest ADI, respectively, across all tested doses. The increase in ADI indicates an enhanced antidiarrheal activity. These findings suggest that the crude extract and solvent fractions of *Colocasia esculenta* (L.) Schott peels possess significant antidiarrheal potential, as evidenced by the notable reduction in key parameters associated with diarrhea.

Overall, the in vivo antidiarrheal activities of the 80% methanol extract and solvent fractions of *Colocasia esculenta* (L.) Schott peels indicate their potential use in managing diarrhea, as demonstrated in the conducted models using Swiss albino mice. These findings highlight the effectiveness of the plant extract as a possible antidiarrheal agent. By considering the different aspects of diarrhea and its associated symptoms, the study provides evidence supporting the utilization of *Colocasia esculenta* (L.) Schott as a natural remedy for diarrhea management.

## 5. Conclusion

The current study demonstrated that both the crude extract and solvent fractions of *Colocasia esculenta* (L.) Schott peel extracts possess significant antidiarrheal activity, likely attributed to their ability to absorb fluids, reduce secretion, and moderately inhibit motility. The evaluation of the in vivo antidiarrheal index indicated that the plant extracts exhibited moderate effects across all tested parameters, with increasing doses showing more pronounced activity. Furthermore, the phytochemical analysis of the plant extracts revealed the presence of various bioactive compounds responsible for the observed antidiarrheal effects. These findings provide scientific evidence supporting the traditional use of *Colocasia esculenta* (L.) Schott in the treatment of diarrhea.

## Figures and Tables

**Table 1:** Results for phytochemical screening of both crude and solvent fractions of peels of *Colocasia esculenta* (L.) Schott.

Secondary metabolites	Methods used	Reagents used	Results			
			CE	AF	CHF	DEEF
Saponins	Foam test	Distilled water	+	+	−	−
Alkaloids	Wagner's test (reddish brown)	Wagner's reagent	+	+	+	−
Flavonoids	Shinoda's test (pink orange)	Ethanol, magnesium chips, concentrated HCl	+	−	+	+
Phenols	Lead acetate test (white precipitate)	Lead acetate solution	+	+	+	−
Tannins	Ferric chloride test (greenish black)	Ferric chloride solution	+	+	−	+
Terpenoids	Salkowski's test (reddish brown)	Chloroform, concentrated H_2_SO_4_	+	+	−	−
Glycosides	Keller–Kiliani test (reddish brown)	Glacial acetic acid, ferric chloride, concentrated H_2_SO_4_	+	−	−	+
Steroids	Salkowski's test (red/green)	Chloroform, concentrated H_2_SO_4_	+	+	−	+
Anthraquinones	Borntrager's test (pink violet/red color)	Chloroform, 10% ammonia solution	+	−	+	+

NB: (+ = presence, − = absence). CE, crude extract; AF, aqueous fraction; CHF, chloroform fraction; DEEF, diethyl ether fraction.

**Table 2:** Effects of the 80% methanol extract on castor oil-induced diarrheal model in mice.

Group	Onset of diarrhea in min	Total number of wet stool over 4 hr	Number of total stool over 4 hr	Total weight of wet stool over 4 hr	The fluid content of the stool	% inhibition of diarrhea	% inhibition of defecation
Negative control	68.67 ± 2.57	10.2 ± 0.48	13.33 ± 0.42	1.63 ± 0.15	0.65 ± 0.09	—	—
80ME 100	71.5 ± 5.7^b1e1^	5.67 ± 0.56^a3b3e1^	7.33 ± 0.88^a3b3e1^	0.91 ± 0.07^a3b2d1e1^	0.29 ± 0.05^a3^	44.03	44.57
80ME 200	98 ± 8.3	4 ± 0.23^a3b1^	6.17 ± 0.48^a3b1^	0.49 ± 0.08^a3c1^	0.15 ± 0.03^a3^	60.58	53.43
80ME 400	142.5 ± 8.2^a2c2^	3.5 ± 0.34^a3c1^	4.83 ± 0.31^a3c1^	0.44 ± 0.03^a3c1^	0.14 ± 0.02^a3^	65.46	63.69
Positive control	137.67 ± 28.05^a1c1^	2.2 ± 0.48^a3c3^	3.67 ± 0.33^a3c3e1^	0.37 ± 0.89^a3c2^	0.11 ± 0.04^a3^	78.23	72.43

Values are expressed by mean ± SEM (*n* = 6), and analysis was performed by one-way ANOVA, followed by post hoc Tukey. Comparisons were made between different groups of study; ^a^compared with negative control; ^b^compared with positive control (loperamide 3 mg/kg); ^c^compared with 80 ME 100; ^d^compared with 80% ME 200; ^e^compared with 80% ME 400; ^1^*P* < 0.05, ^2^*P* < 0.01, and ^3^*P* < 0.001. Negative controls received 2% Tween 80; 80 ME 100 = 100 mg/kg of 80% methanol extract; 80 ME 200 = 200 mg/kg of 80% methanol extract; 80 ME 400 = 400 mg/kg of 80% methanol extract.

**Table 3:** Effects of the 80% methanol extract on castor oil-induced enteropooling model in mice.

Group	Mean weight of small intestinal content in (g)	% inhibition by weight (%)	Mean volume of small intestinal content in (mL)	% inhibition by volume (%)
Negative control	0.62 ± 0.07	—	0.53 ± 0.05	—
80 ME 100	0.38 ± 0.04^a2^	34.5	0.3 ± 0.01^a3b2d1^	41
80 ME 200	0.36 ± 0.05^a2^	42.3	0.23 ± 0.04^a3c1^	57
80 ME 400	0.23 ± 0.03^a3^	60.4	0.17 ± 0.02^a3c1^	66.4
Positive control	0.21 ± 0.23^a3^	65.7	0.14 ± 0.01^a3c2^	72.4

Values are expressed as mean ± SEM (*n* = 6), and analysis was performed using one-way ANOVA, followed by post hoc Tukey; ^a^compared with negative control (2% Tween 80), ^b^compared with positive control (loperamide 3 mg/kg); ^c^compared with 80 MW 100; ^d^compared with 80 ME 400; ^1^*P* < 0.05, ^2^*P* < 0.01, and ^3^*P* < 0.001. 80 ME 100 = 100 mg/kg of 80% methanol extract; 80 ME 200 = 200 mg/kg of 80% methanol extract; 80 ME 400 = 400 mg/kg of 80% methanol extract.

**Table 4:** Effects of the 80% methanol extract on castor oil-induced gastrointestinal motility model in mice.

Group	Length of the small intestine in (cm)	Distance traveled by charcoal in (cm)	Peristalsis index (%)	% inhibition (%)
Negative control	57.4 ± 1.5	49 ± 1.6	85.6 ± 2.3	—
80 ME 100	55.7 ± 1.7	39.83 ± 1.22^a2b3e2^	71.9 ± 3.36^a1b3e3^	18.2
80 ME 200	58 ± 1.5	37.83 ± 2.12^a3b3e1^	65.15 ± 3.1^a3b3e1^	22.3
80 ME 400	57.8 ± 1.4	30.1 ± 1.8^a3b3c2d1^	52±3^a3b3c3d1^	38.9
Positive control	58.7 ± 1.2	17.16 ± 1.24^a3c3d3e3^	29.1 ± 1.7^a3c3d3e3^	64.4

Data are expressed as mean ± SEM (*n* = 6), and analysis was performed using one-way ANOVA, followed by post hoc Tukey; ^a^compared with negative control (2% Tween 80), ^b^compared with positive control (atropine 5 mg/kg); ^c^compared with 80 MW 100; ^d^compared with 80 ME 200; ^e^compared with 80 ME 400; ^1^*P* < 0.05, ^2^*P* < 0.01, and ^3^*P* < 0.001. 80 ME 100 = 100 mg/kg of 80% methanol extract; 80 ME 200 = 200 mg/kg of 80% methanol extract; 80 ME 400 = 400 mg/kg of 80% methanol extract.

**Table 5:** Effects of solvent fractions on castor oil-induced diarrheal model.

Group	Delay of diarrheal onset in min	Total number of wet stool over 4 hr	Number of total stool over 4 hr	Total weight of wet stool over 4 hr	The fluid content of the stool	% inhibition of diarrhea	% inhibition of defecation
Negative control	66.83 ± 3.3	9.8 ± 0.5	13 ± 0.58	1.58 ± 0.15	0.55 ± 0.04	—	—
AF 100	59.8 ± 3.89^b3e3h3k3^	6.67 ± 0.33^a3b3e2h2k2^	8.5 ± 0.2^a3b3e3h3k2^	1.2 ± 0.09^b3e3h2k1^	0.4 ± 0.052^b2^	31.67	33.89
AF 200	76.83 ± 4.79^b3e1h3k2^	5.33 ± 0.2^a3b2i1^	7.17 ± 0.4^a3b3e2h3i2^	0.97 ± 0.1^a2b1i1^	0.39 ± 0.1^b1^	44.86	44.5
AF 400	109.5 ± 8.54^a3b3c3d1f3i3j3^	4.33 ± 0.2^a3c2f3i3j1^	5 ± 0.3^a3c3d2f3g2i3j3^	0.58 ± 0.07^a3c2f3i3j1^	0.26 ± 0.06^a2f1i1^	55	61.4
CHF100	66 ± 4.2^b3e3h3k3^	7 ± 0.45^a3b3e3h3k2^	8 ± 0.26^a3b3e3h3k1^	1.3 ± 0.09^b3e3h3k2^	0.53 ± 0.05^b3e1h1k1^	30.75	28.58
CHF 200	85.2 ± 5.58^b3h2k1^	5.83 ± 0.5^a3b3^	7.33 ± 0.67^a3b3e2h3i2^	0.99 ± 0.11^a2b1i1^	0.37 ± 0.04^b1^	40.85	35.62
CHF 400	119.2 ± 7.55^a3b3c3d3f3g2i3j3^	4.2 ± 0.5^a3c2f3i3j2^	4.5 ± 0.2^a3c3d3f3g3i3j3^	0.57 ± 0.07^a3c2f3i3j1^	0.25 ± 0.02^a2f1i1^	57.73	38.34
DEEF 100	54.33 ± 3.45^b3e3h3k3^	7.33 ± 0.33^a3b3d2e3h3k3^	9.5 ± 0.22^a3b3d2e3g2h3k3^	1.5 ± 0.14^b3d1e3g1i3k3^	0.53 ± 0.06^b3e1h1k1^	27.5	28.44
DEEF200	68.83 ± 4.63^b3e3h3k3^	6.33 ± 0.42^a3b3e2h2^	8.83 ± 0.3^a3b3e3h3k3^	1.1 ± 0.1^b2e1h1^	0.36 ± 0.05^b1^	37.23	33.22
DEEF 400	113.5 ± 9.65^a3b3c3d2f3g1i3j3^	4.5 ± 0.34^a3f2i3^	6 ± 0.3^a3b2c2f1i3j3^	0.66 ± 0.1^a3c1f2i3^	0.26 ± 0.03^a1f1i1^	55.94	54.59
Loperamide	144 ± 4.85^a3c3d3e3f3g3h3i3j3k3^	2.83 ± 0.3^a3cd2f3g3i3j3^	3.67 ± 0.42^a3c3d3f3g3i3j3^	0.44 ± 0.05^a3c3d1f3g1i3j2^	0.1 ± 0.02^a3c2d1f3g1i3j1^	70.69	71

Data are expressed by mean ± SEM (*n* = 6); analysis was done by one-way ANOVA, followed by post hoc Tukey. ^a^Compared with negative control (2% Tween 80); ^b^compared with positive control (loperamide 3 mg/kg); ^c^compared with 100 mg/kg AF; ^d^compared with 200 mg/kg AF; ^e^compared with 400 mg/kg AF; ^f^compared with 100 mg/kg CHF; ^g^compared with 200 mg/kg CHF; ^h^compared with 400 mg/kg CHF; ^i^compared with 100 mg/kg DEEF; ^j^compared with 200 mg/kg DEEF; ^k^compared with 400 mg/kg DEEF; ^1^*P* < 0.05, ^2^*P*<0.01, and ^3^*P*<0.001. AF, aqueous fraction; CHF, chloroform fraction; DEEF, diethyl ether fraction.

**Table 6:** The effects of solvent fractions on castor oil-induced enteropooling model in mice.

Group	Mean weight of small intestinal content in (g)	% inhibition by weight (%)	Mean volume of small intestinal content in (mL)	% inhibition by volume (%)
Negative control	0.69 ± 0.06	—	0.52 ± 0.04	—
AF 100	0.5 ± 0.06^a2b3e2h2k3^	29.68	0.36 ± 0.04^a1b3e1k2^	31.2
AF 200	0.38 ± 0.03^a3^	45.36	0.27 ± 0.04^a3^	49.2
AF 400	0.28 ± 0.03^a3c2f1^	58.9	0.2 ± 0.03^a3c1f2^	61.7
CHF 100	0.46 ± 0.04^a2b3e1h1k2^	22.2	0.4 ± 0.04^b3e2k3^	22.38
CHF 200	0.35 ± 0.03^a3^	39.6	0.3 ± 0.03^a2b1^	41.22
CHF 400	0.26 ± 02^a3c2f2i1^	53.78	0.24 ± 0.03^a3^	52.7
DEEF 100	0.44 ± 0.03^a3b2h1k2^	26.7	0.33 ± 0.04^a2b2k1^	27
DEEF 200	0.36 ± 0.021^a3^	41.2	0.25 ± 0.03^a3^	43
DEEF 400	0.24 ± 0.01^a3c3f2i2^	60.2	0.2 ± 0.02^a3c2f3i1^	62.6
Loperamide	0.21 ± 0.02^a3c3f3i2^	68.8	0.14 ± 0.01^a3c3f3g1i2^	73.7

Values are expressed by mean ± SEM (*n* = 6); analysis was performed by one-way ANOVA, followed by post hoc Tukey. Comparisons were made between different groups of study; ^a^compared with negative control; ^b^compared with positive control (loperamide 3 mg/kg). ^c^Compared with 100 mg/kg AF; ^d^compared with 200 mg/kg AF; ^e^compared with 400 mg/kg AF; ^f^compared with 100 mg/kg CHF; ^g^compared with 200 mg/kg CHF; ^h^compared with 400 mg/kg CHF; ^i^compared with 100 mg/kg DEEF; ^j^compared with 200 mg/kg DEEF; ^k^compared with 400 mg/kg DEEF; ^1^*P* < 0.05, ^2^*P* < 0.01, and ^3^*P* < 0.001. AF, aqueous fraction; CHF, chloroform fraction; DEEF, diethyl ether fraction. NB: negative control 2% Tween 80 for all fractions.

**Table 7:** The effects of the solvent fractions on castor oil-induced gastrointestinal motility model in mice.

Group	Length of the small intestine in (cm)	Distance traveled by charcoal in (cm)	Peristalsis index (%)	% inhibition (%)
Negative control	53.9 ± 1.7	46.5 ± 2.25	86 ± 2	—
AF 100	58.3 ± 0.86	45.67 ± 1.28^b3e3g1h3k1^	78.28 ± 1.8^b3e3h3k3^	9
AF 200	54.3 ± 2.03	38.77 ± 1.49^b3h1^	71.59 ± 2.45^a2b3e2h3^	16.8
AF 400	56.75 ± 2.09	33.43 ± 3.26^a3b3f2i3^	58.4 ± 4.2^a3b3c3d2e3f2i3j2^	31.8
CHF 100	58.68 ± 1.46	44.9 ± 1.12^b3e2h3k1^	76.6 ± 1.2^b3e3h3k2^	7.43
CHF 200	51.83 ± 1.39	37 ± 1.54^a1b3c1i1^	71.59 ± 3.25^a2b3e2h3^	13.63
CHF 400	55.42 ± 2.42	30.55 ± 1.54^a3b3c3d1f3i3j1^	55.2 ± 1.8^a3b3c3d3f3g3i3j3^	33.2
DEEF 100	58.28 ± 1	45.68 ± 0.93^b3e3g1h3k1^	78.4 ± 1.2^b3e3h3k3^	6.8
DEEF 200	52.57 ± 1.5	38.65 ± 1.2^b3h1^	73.5 ± 0.7^b3e2h3k1^	12.6
DEEF 400	59.45 ± 0.88	36.5 ± 1.6^a2b3c1f1i1^	61.3 ± 2.3^a3b3c3f2i3j1^	27
Atropine	57.8 ± 1.3	17.42 ± 1.23^a3c3d3e3f3g3h3i3j3k3^	30 ± 1.77^a3c3d3e3f3g3i3j3k3^	65

Data are expressed by mean ± SEM (*n* = 6); analysis was conducted by one-way ANOVA, followed by post hoc Tukey. Comparisons were made between different groups of study; ^a^compared with negative control; ^b^compared with positive control (atropine 5 mg/kg). ^c^Compared with 100 mg/kg AF; ^d^compared with 200 mg/kg AF; ^e^compared with 400 mg/kg AF; ^f^compared with 100 mg/kg CHF; ^g^compared with 200 mg/kg CHF; ^h^compared with 400 mg/kg CHF; ^i^compared with 100 mg/kg DEEF; ^j^compared with 200 mg/kg DEEF; ^k^compared with 400 mg/kg DEEF; ^1^*P* < 0.05, ^2^*P* < 0.01, and ^3^*P* < 0.001. AF, aqueous fraction; CHF, chloroform fraction; DEEF, diethyl ether fraction. NB: negative control 2% Tween 80 for all fractions.

## Data Availability

Most of the data are included in the manuscript. Additional data can be found from the corresponding author based upon reasonable request.

## Abbreviations

ADI: In vivo antidiarrheal Index
ATCC: American type culture collection
CaCC: Calcium-activated chloride channels
cAMP: Cyclic adenosine monophosphate
CFTR: Cystic fibrosis transmembrane conductance regulator
CLSI: Clinical and laboratory standard institution
COX: Cyclooxygenase
DAG: Diacylglycerol
DDT: The delay in defecation time or diarrheal onset
DMSO: Dimethyl sulfoxide
DRA: Downregulated in adenoma
EDHS: Ethiopian demographic and health surveys
ENaC: Epithelial sodium channel
EPEC: Enteropathogenic *Escherichia coli*
EPHI: Ethiopian Public Health Institute
ETEC: Enterotoxigenic *Escherichia coli*
GT: The gastrointestinal motility by charcoal travel reduction
KCNN4: Potassium calcium-activated channel subfamily N member 4
LOX: Lipoxygenase
NHE: Sodium hydrogen exchange
NHERF1: Na + /H + exchange regulatory factor 1
NKCC1: Sodium potassium chloride cotransporter 1
OECD: Organization for Economic Co-operation and Development
PAF: Platelet-activating factor
PCR: Polymerase chain reaction
PG: Prostaglandin
PI: Peristalsis index.
